# Small Molecule Suppressors of *Drosophila* Kinesin Deficiency Rescue Motor Axon Development in a Zebrafish Model of Spinal Muscular Atrophy

**DOI:** 10.1371/journal.pone.0074325

**Published:** 2013-09-04

**Authors:** Andrew Gassman, Le T. Hao, Leena Bhoite, Chad L. Bradford, Chi-Bin Chien, Christine E. Beattie, John P. Manfredi

**Affiliations:** 1 Sera Prognostics, Inc., Salt Lake City, Utah, United States of America; 2 Department of Neuroscience, The Ohio State University, Columbus, Ohio, United States of America; 3 Technology Commercialization Office, University of Utah, Salt Lake City, Utah, United States of America; 4 Department of Neurobiology and Anatomy, University of Utah, Salt Lake City, Utah, United States of America; 5 Sfida BioLogic, Inc., Salt Lake City, Utah, United States of America; University of Edinburgh, United Kingdom

## Abstract

Proximal spinal muscular atrophy (SMA) is the most common inherited motor neuropathy and the leading hereditary cause of infant mortality. Currently there is no effective treatment for the disease, reflecting a need for pharmacologic interventions that restore performance of dysfunctional motor neurons or suppress the consequences of their dysfunction. In a series of assays relevant to motor neuron biology, we explored the activities of a collection of tetrahydroindoles that were reported to alter the metabolism of amyloid precursor protein (APP). In *Drosophila* larvae the compounds suppressed aberrant larval locomotion due to mutations in the *Khc* and *Klc* genes, which respectively encode the heavy and light chains of kinesin-1. A representative compound of this class also suppressed the appearance of axonal swellings (alternatively termed axonal spheroids or neuritic beads) in the segmental nerves of the kinesin-deficient *Drosophila* larvae. Given the importance of kinesin-dependent transport for extension and maintenance of axons and their growth cones, three members of the class were tested for neurotrophic effects on isolated rat spinal motor neurons. Each compound stimulated neurite outgrowth. In addition, consistent with SMA being an axonopathy of motor neurons, the three axonotrophic compounds rescued motor axon development in a zebrafish model of SMA. The results introduce a collection of small molecules as pharmacologic suppressors of SMA-associated phenotypes and nominate specific members of the collection for development as candidate SMA therapeutics. More generally, the results reinforce the perception of SMA as an axonopathy and suggest novel approaches to treating the disease.

## Introduction

SMA results from inadequate levels of the ubiquitously expressed protein SMN [1]. Given the expression of SMN throughout the body, it is paradoxical that its deficiency preferentially affects motor neurons in the anterior horn of the spinal cord [2]. This suggests that some distinguishing and essential feature of spinal motor neurons is particularly vulnerable to SMN deficiency. One such feature is the neuromuscular junction – a highly specialized structure that develops where the motor axon terminates on muscle and that is dependent on the proper function of the distal motor axon and its terminal [3]. It is possible, then, that a primary consequence of SMN deficiency is dysfunction of distal motor axons and their terminals. Indeed, SMN deficiency is reported to disrupt processing of pre-mRNAs encoding subunits of kinesin and dynein (*viz*., Kif17, Klc4, and Dync1h1) [4], which drive transport of diverse cargoes in axons [5]. The function and viability of distal axons and terminals of spinal motor neurons, given their exceptional lengths, are particularly dependent on the activities of these motors. Another transcript that suffers disrupted processing as a result of SMN deficiency encodes Stasimon [6], whose resulting deficiency alters neurotransmitter release at motor axon terminals [7]. Inadequate SMN levels also compromise the formation of mRNPs, complexes that regulate mRNA transport, stability, and local translation in axons. The consequently reduced levels of the encoded proteins in axons and their growth cones can severely affect the function of distal axons and their terminals [8]–[10]. Yet other mechanisms by which reduced SMN levels can adversely affect axonal function involve the interaction of SMN with both plastin 3, which promotes axonogenesis via its effects on actin [11], [12], and profilin II, which influences growth cone motility via its effects on actin and ROCK [13]–[22]. Through all of these disparate mechanisms, low SMN levels can compromise the function of the distal motor axon and its terminal and thereby contribute to SMA pathology. Accordingly, agents that enhance the growth, development, and performance of motor axons *(i.e.*, axonotrophic agents) may be therapeutic for SMA.

We considered the possibility that such axonotrophic agents may be found among compounds that affect the processing of amyloid precursor protein (APP). This hypothesis is based on the well-founded association between APP processing and intracellular membrane trafficking [23], [24]. For example, both endocytic and secretory events have been shown to influence the proteolytic fate of APP [25]–[30]. In addition, the enzymes that process APP affect its trafficking and the distribution of its proteolytic fragments among different cellular compartments [31]–[33]. Thus, the profile of proteolytic fragments of APP, their locations within the cell, and the likelihoods of their extracellular release are tightly coupled to intracellular membrane trafficking. The association between APP processing and intracellular membrane trafficking suggests that molecular motors such as kinesin may play a role in APP metabolism. Indeed, kinesin deficiency in mice is reported to alter γ-secretase-mediated processing of APP [34]. Specifically, deletion of one copy of the kinesin light chain increased the levels of the APP-derived peptides Aβ42 and Aβ40 in brains of mice that express APP mutants associated with Alzheimer’s disease. Thus, kinesin function and metabolism of Aβ peptides may be mechanistically coupled, suggesting that agents that modulate Aβ metabolism may affect kinesin function. Given the importance of kinesin for the growth, development, and function of motor axons [35], these observations raise the possibility that agents that modulate Aβ metabolism can have axonotrophic activities.

**Figure 1:**
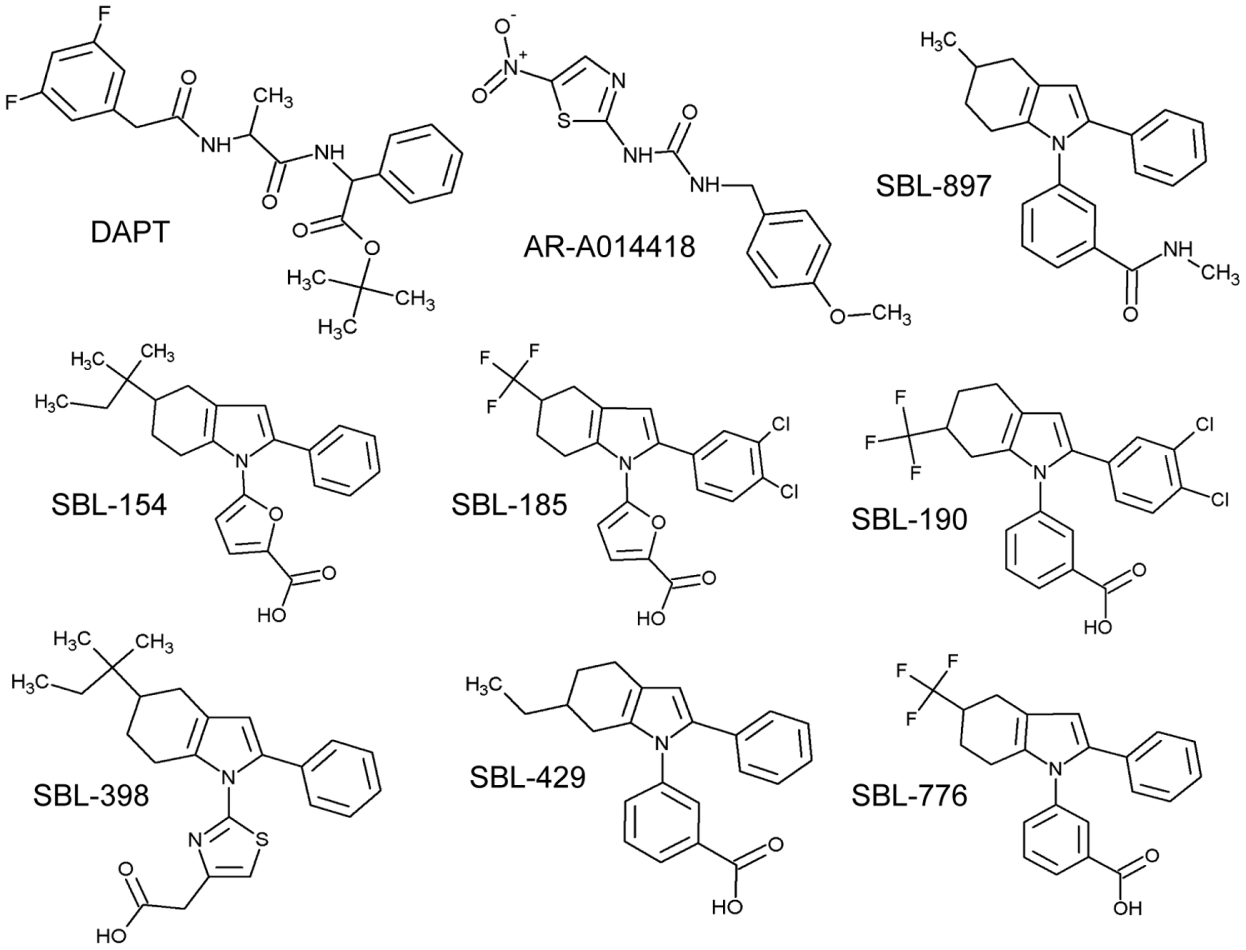
Structures of small molecules used in this study. The six compounds that exhibit positive effects in this study (*viz*., SBL-154, SBL-185, SBL-190, SBL-398, SBL-429, and SBL-776) are new chemical entities that were disclosed in a recently issued patent as agents that lower production of Aβ42 [36]. SBL-897, an analog of the active compounds, showed no effect on Aβ42 production and no effects in this study. These compounds have not previously been reported in the scientific literature. DAPT is a γ-secretase inhibitor, and AR-A014418 is a commercial GSK3β inhibitor.

We explored this possibility using structurally related small molecules that have been reported in the patent literature to alter Aβ metabolism [36] (Fig. 1). Specifically, the compounds were disclosed as inhibiting production of Aβ42 by cultured H4 neuroglioma cells engineered to overexpress APP. We report here that these compounds rescue normal locomotion of kinesin-deficient *Drosophila* larvae and promote neurite outgrowth of isolated spinal motor neurons. We further report that these axonotrophic compounds rescue motor axon development in Smn-deficient zebrafish. The findings recommend novel approaches to the development of SMA therapeutics.

## Results

### Reported Inhibitors of Aβ42 Production Suppress Kinesin Deficiency in *Drosophila* Larvae

**Figure 2:**
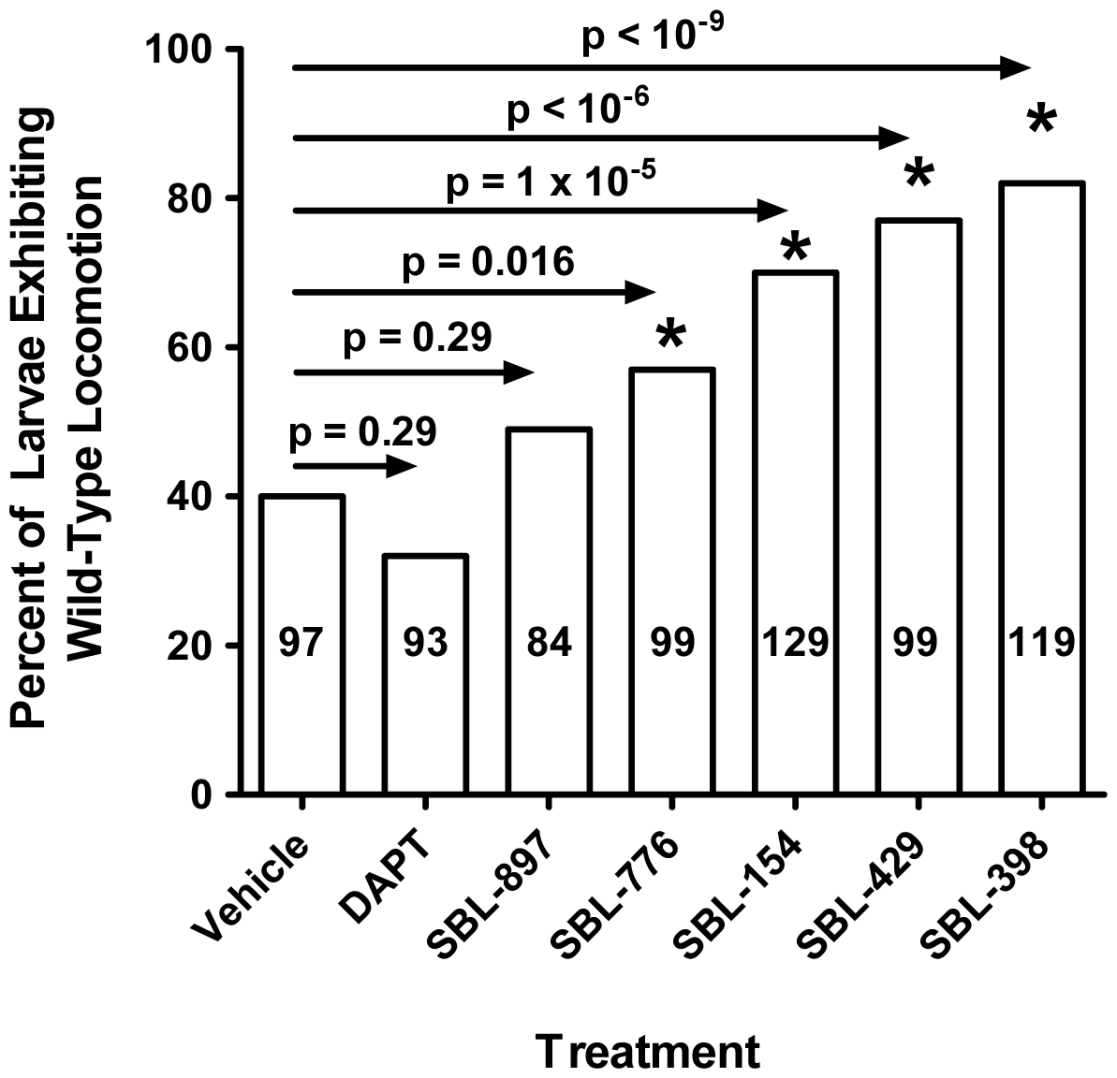
Pharmacologic suppression of locomotion defect of kinesin-deficient *Drosophila*. *khc/+; klc/+* larvae were grown in the presence of vehicle alone (DMSO) or 0.5 mM of the indicated compounds and scored for the characteristic tail-flipping phenotype displayed by kinesin-deficient larvae. The number of larvae scored for each treatment is indicated within the relevant bar. Results are expressed in terms of the percentage of larvae that move normally (*i.e.*, do not exhibit uncoordinated locomotion). Thus, the extent of suppression of motor dysfunction is reflected by the heights of the bars relative to DMSO treatment. Since no wild-type larva was seen to display the tail-flipping phenotype (data not shown), the percentage for the vehicle condition (40%) indicates 60% penetrance of the phenotype. p values, calculated by application of Fisher’s exact test to each experimental condition *vs.* DMSO, are shown. With the sequential Bonferroni method [120], [121] to determine significance (*) at α = 0.05 (thereby accommodating multiple testing issues), the results indicate with 95% confidence that SBL-154, SBL-429, SBL-398, and SBL-776 rescue coordinated locomotion.

Deletion of one copy of the kinesin light chain was reported to increase the level of APP-derived Aβ peptides in brains of mice that express APP mutants associated with Alzheimer’s disease [34]. This association of kinesin function with production of Aβ peptides admits the possibility that modulators of Aβ production may modify kinesin-dependent phenotypes such as the abnormal locomotion of kinesin-deficient *Drosophila* larvae. To evaluate this possibility, we tested six compounds for rescue of locomotion of *khc/+; klc/+* larvae, which lack one copy of the genes encoding the heavy and light chains of kinesin-1. Of the six tested compounds, four (SBL-154, SBL-429, SBL-398, and SBL-776) were reported in the patent literature to lower Aβ42 production [36]; one compound (SBL-897) showed no effect on Aβ production; and one compound (DAPT) has been shown to inhibit secretion of all Aβ peptides in several mammalian systems [37]–[39]. Neither SBL-897 nor DAPT significantly affected the fraction of mutant larvae with motor dysfunction (Fig. 2). In contrast, each of the compounds disclosed to lower Aβ42 production rescued locomotion of the mutant larvae (Fig. 2). Notably, the tested concentration of DAPT is equivalent to concentrations that have previously been reported to alter Notch-dependent phenotypes in *Drosophila* [40], [41].

**Figure 3:**
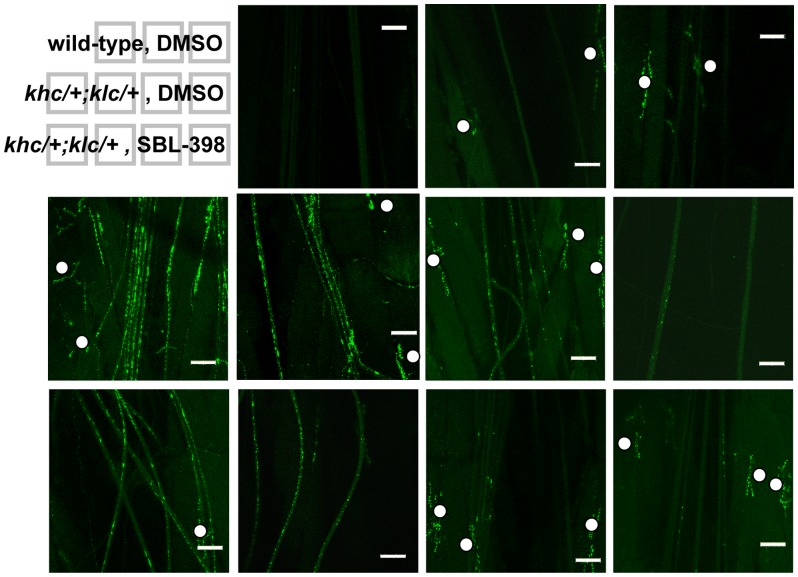
Reduction of neuronal aggregates by SBL-398; representative results. Eleven images, each representing a different larva, depict the range of phenotypes observed for: wild-type larvae treated with DMSO (top row); *khc/+; klc/*+ larvae treated with DMSO (middle row); and *khc/+; klc/*+ larvae treated with 0.5 mM SBL-398 (bottom row). Central images on each row represent the most commonly observed phenotypes for each condition. Solid dots demarcate synaptic boutons, which are stained by anti-synaptogamin but should not be confused with intraneuronal aggregates. The genuine aggregates are distinguished by their distribution throughout the lengths of segmental nerves that have a generally longitudinal orientation. White bars represent 50 µm.

**Figure 4:**
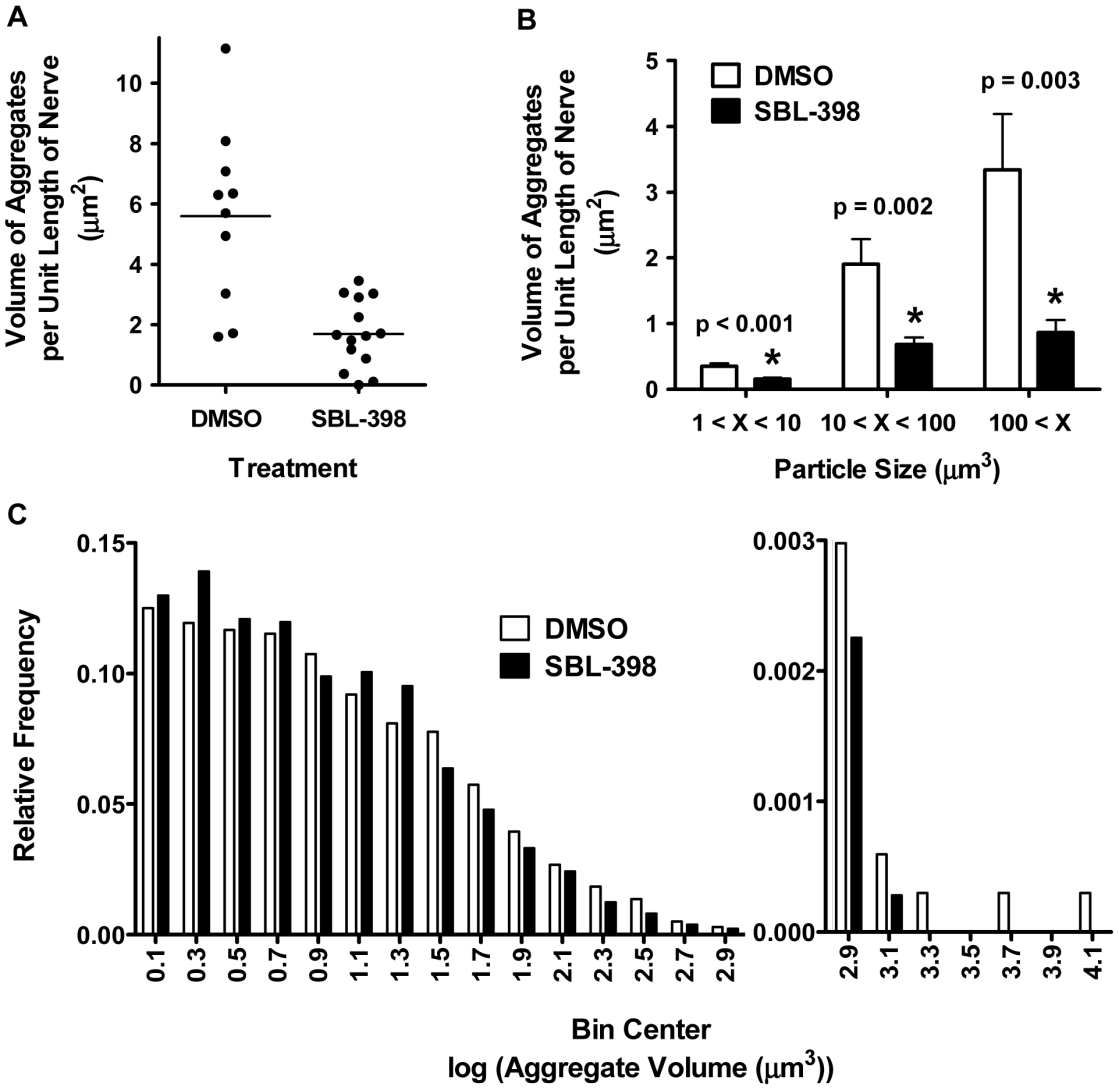
Reduction of neuronal aggregates by SBL-398; combined results. (A) Volumes of aggregates per unit length of nerve are plotted for *khc/+; klc/*+ larvae raised on media containing DMSO (N = 10 larvae) or 0.5 mM SBL-398 (N = 14 larvae). Each data point represents one animal. The large variance in the DMSO condition likely reflects incomplete penetrance (typical penetrance is about 0.7). Despite this variance, the mean values differ significantly (p<0.001, unpaired t test). (B) Mean volumes of aggregates per unit length of nerve are shown for three classes of aggregate sizes in DMSO-treated (N = 10; white bars) and SBL-398-treated (N = 14; black bars) larvae. Aggregate volumes per length of nerve are significantly (*) lower in SBL-398-treated larvae regardless of size class (t test with Bonferroni method to determine significance [120], [121]). (C) For both DMSO- and SBL-398-treated animals, aggregates greater than 1 µm^3^ were distributed among 0.2 log unit-wide bins. The frequency distribution of the aggregates is shown for DMSO-treated (white bars; 3,359 objects) and SBL-398-treated larvae (black bars; 3,551 objects). The differently scaled y-axis for bins containing the largest aggregates allows visual discrimination of histograms throughout the entire range of aggregate volumes. SBL-398 significantly shifts the size distribution of aggregates to smaller volumes (Mann Whitney test; p<0.001).

The aberrant locomotion of kinesin mutants is highly correlated with the accumulation in axons of membranous debris derived from vesicles, mitochondria, synaptic membranes, and pre-lysosomal organelles [35], [42]. We predicted that compounds that suppress the locomotion defect would also suppress the appearance of these membranous aggregates. One compound, SBL-398, was chosen to test this prediction. Treatment of larvae with SBL-398 significantly reduced the total volume of axonal aggregates to 30% of the level seen in vehicle-treated controls (Figs. 3, 4A). Reduction of the mass of membranous accumulations was not restricted to a particular size of aggregate. Thus, when aggregates were classified as small (1 µm^3^<volume<10 µm^3^), medium (10 µm^3^<volume<100 µm^3^), or large (100 µm^3^<volume), reductions were observed for all three classes (Fig. 4B). This effect of SBL-398 is also indicated by a significant compound-induced shift in the frequency distribution of aggregate sizes towards smaller values (Fig. 4C).

The high levels of compounds in the medium on which larvae are raised (*viz*., 0.5 mM) are typical of *Drosophila* studies that explore drug effects [41], [43]–[50]. Still, it is possible that the levels of compounds used in our experiments result in extraordinarily high concentrations in the animals. To address this possibility, we assayed by LC-MS/MS the levels of SBL-398 in hemolymph of larvae raised on media containing 0.5 mM compound. This concentration of SBL-398 suppressed motor dysfunction in 69% of *khc/+; klc/+* larvae (Fig. 2) and reduced by 70% the mass of membranous aggregates in their segmental nerves (Fig. 4A). No trace of SBL-398 was observed in hemolymph of larvae treated with DMSO vehicle, indicating negligible background signal in the LC-MS/MS assay. The concentration of SBL-398 in hemolymph of compound-treated larvae averaged 228 nM; concentrations in the independently collected duplicate samples were 207 and 248 nM. That is, the concentration in hemolymph was less than 1/2,000^th^ the nominal concentration in the media. There existed an apparent metabolite of SBL-398 of greater molecular weight, suggesting modification (*e.g.,* oxidation) of the parent compound. The signal from this single metabolite was equivalent to SBL-398. We conclude that the suppressor effects of SBL-398 and, by inference, its analogs are observed at pharmacologically reasonable concentrations of compounds.

### GSK-3 is not the Compounds’ Target

**Figure 5:**
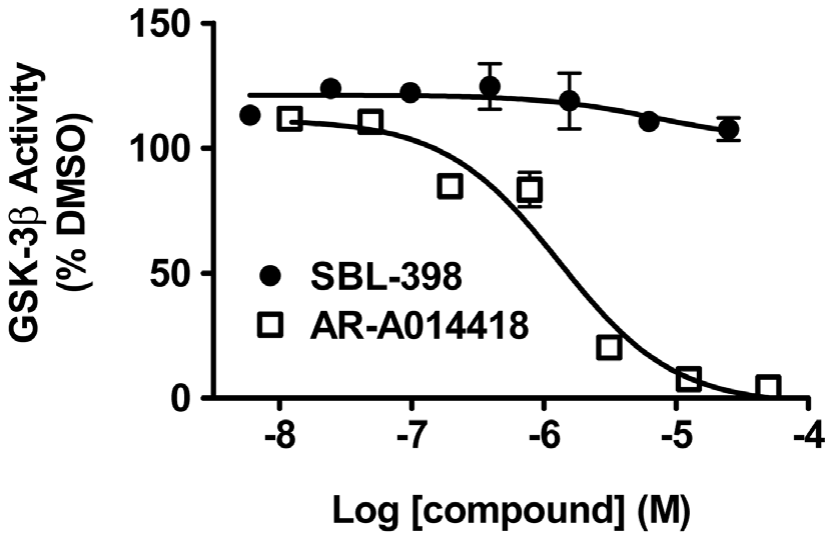
Lack of inhibition of human GSK-3β by SBL-398. Enzymatic activity of purified human GSK-3β, assayed by *in vitro* phosphorylation of a peptide substrate (see Methods), was not inhibited by concentrations of SBL-398 that approached its aqueous solubility limit. Complete inhibition was achieved with AR-A014418, a commercially available GSK-3β inhibitor. Data points are averages of duplicate samples; apparently absent error bars are in fact obscured by the data symbols.

It has recently been reported that the motor dysfunction of kinesin-deficient *Drosophila* and the accumulation of axonal aggregates are mitigated by experimental reductions in the activity of GSK-3 [51]. It is possible, then, that the pharmacologic suppressors of *khc/+; klc/+* (*viz*., SBL-154, SBL-429, SBL-398, and SBL-776,) achieve their effects by inhibiting GSK-3. This possibility is reinforced by reports that mammalian GSK-3β restrains kinesin-mediate axonal transport of diverse cargoes [49], [50], [52]–[55]. Thus, in a kinesin-mutant background where anterograde transport is genetically compromised, endogenous GSK-3 activity could disproportionately reduce already-diminished kinesin-mediated transport. Accordingly, pharmacologic reduction of GSK-3 activity could restore sufficient transport to suppress the effects of kinesin deficiency. We evaluated this possibility by testing the inhibitory potency of SBL-398 on purified human GSK-3β. The human protein used is 73–79% identical and 85–88% similar to the 8 isoforms of Shaggy, the GSK-3β ortholog in *Drosophila*. No inhibition of the human GSK-3β was observed at concentrations as high as 25 µM (Fig. 5), which is more than 100-fold higher than the concentration in hemolymph of treated larvae. In contrast, complete inhibition was obtained with the known GSK-3β inhibitor AR-A014418 [56], with an IC_50_ of 1.3±0.4 µM. We conclude that inhibition of GSK-3 does not account for SBL-398’s ability to suppress the axonal swellings and aberrant locomotion of *khc/+; klc/+* larvae.

### The Compounds Stimulate Neurite Outgrowth of Spinal Motor Neurons

SBL-398 is a member of a collection of novel, structurally related molecules that alter Aβ metabolism [36]. The four members of this collection that were tested for suppression of kinesin deficiency in *Drosophila* larvae all showed positive effects (Fig. 2), suggesting that the compounds affect anterograde transport in motor axons. Since extension and maintenance of axons and their growth cones depend on anterograde transport of membrane-bound organelles, protein complexes, and mRNA-containing particles [57], [58], we hypothesized that members of the collection would promote neurite outgrowth of motor neurons. We tested this hypothesis using motor neurons isolated from embryonic rat spinal cords.

**Figure 6:**
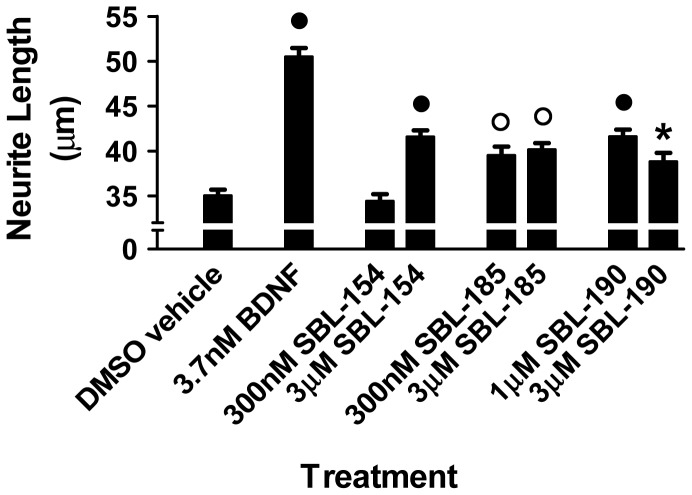
Stimulation of neurite outgrowth in rat spinal motor neurons. Primary cultures of rat embryonic spinal motor neurons were treated with BDNF at 3.7 nM and with SBL-154, SBL-185, and SBL-190 at the two indicated concentrations of each compound. One-way ANOVA with Dunnett’s post test indicates that the positive control BDNF and each compound significantly enhanced neurite outgrowth relative to DMSO vehicle (N = 3 cultures; • p<0.001; **○** p<0.01; * p<0.05).

Of the 4 molecules that were shown to suppress the locomotion defect of kinesin-deficient *Drosophila* larvae, one (SBL-154) exhibited superior PK (pharmacokinetic) and ADMET (absorption, distribution, excretion, metabolism, toxicity) properties in rats and mice (data not shown). Because of its attractive PK and ADMET properties, we further evaluated SBL-154 by examining the compound’s effects on neurite outgrowth and survival of rat spinal motor neurons. We included two structural analogs of SBL-154 that, like SBL-154, exhibit attractive PK and ADMET properties (data not shown) and are reported to inhibit Aβ42 production [36]. Motor neurons isolated from spinal cords of E15 rat embryos were cultured for 3 days in the presence of the compounds and morphometrically analyzed to quantify neurite lengths. All three compounds significantly stimulated neurite outgrowth (Fig. 6). Using the same concentrations of the three compounds, we also assayed their effects on survival of the isolated rat motor neurons. Whereas the positive control, BDNF, consistently increased survival of three independent motor neuron preparations (21±8%, mean increase ± SD, N = 3), none of the three experimental compounds improved survival consistently, resulting in no significant effects (data not shown). Notably, a reduction in survival was never observed. Thus, the compounds stimulated motor axon outgrowth as determined by neurite length without affecting motor neuron survival.

### The Compounds Rescue Development of Motor Axons in a Zebrafish SMA Model

SMA is caused by low levels of the survival motor neuron protein (SMN) [1], which can be modeled in zebrafish using antisense morpholino (MO) to decrease Smn protein levels [59]. MO-mediated reduction of zebrafish Smn levels results in motor axon defects such as truncation and aberrant branching, defects that are rescued by heterologous expression of human SMN. Such results indicate that the motor axon defects are a read-out of low Smn levels and indicate that motor neuron development is abnormal when Smn levels are low [59]–[61]. To evaluate the effects of these small molecules on motor axon development in Smn-deficient embryos, we applied a previously published scoring algorithm in which individual embryos are classified according to the number of defective motor axons and the severity of the defects [60]. Thus, an embryo is classified as severe, moderate, mild, or unaffected on the basis of its motor axon defects [8].

**Figure 7:**
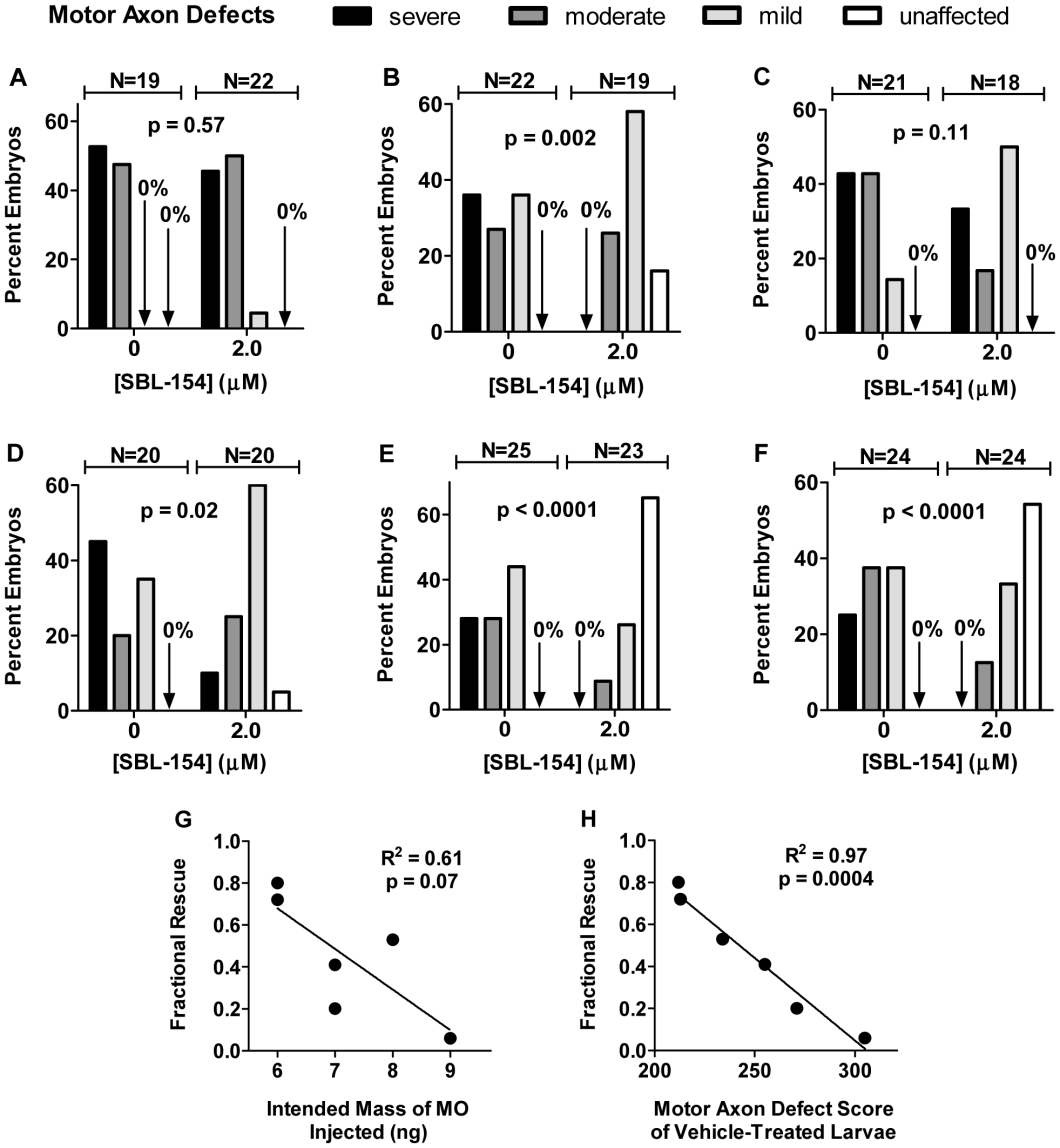
Rescue of motor axon development in Smn-deficient zebrafish by SBL-154. (A-F) Zebrafish embryos injected with *smn* MO and treated with either DMSO vehicle or 2 µM SBL-154 were classified by degree of motor axon defects. Distributions of embryos among the 4 classes of severity of motor axon dysmorphism are shown for the vehicle and SBL-154 conditions for each of 6 experiments. The p values comparing the two distributions were calculated for the individual experiments by the Mann-Whitney U test and are indicated in each graph along with the numbers of vehicle- and compound-treated embryos scored in that experiment. For any single embryo, approximately 20 axons were evaluated, resulting in about 400 axons scored for each of the control and experimental conditions in each experiment. As indicated in the text, the nominal mass of *smn* MO injected varied among experiments: A: 9 ng; B: 8 ng; C: 7 ng; D: 7 ng; E: 6 ng; F: 6 ng. (G & H) Suppression of motor axon abnormalities in embryos injected with *smn* MO by 2 µM SBL-154 is expressed in terms of Fractional Rescue, as described in Materials and Methods. The Fractional Rescues for the 6 individual experiments shown in panels A-F are plotted as a function of two different measures of the amount of *smn* MO delivered to the embryos: (G) the nominal, intended mass of injected MO or (H) the Motor Axon Defect score, a metric of the severity of motor axon abnormalities, of vehicle-treated embryos (see Materials and Methods). Parameters of the linear regressions in each graph are indicated. The two experiments associated with the lowest Fractional Rescues (values of 0.2 and 0.06, corresponding to panels A and C, respectively) failed to show significant rescue of motor axon development by SBL-154.

**Figure 8:**
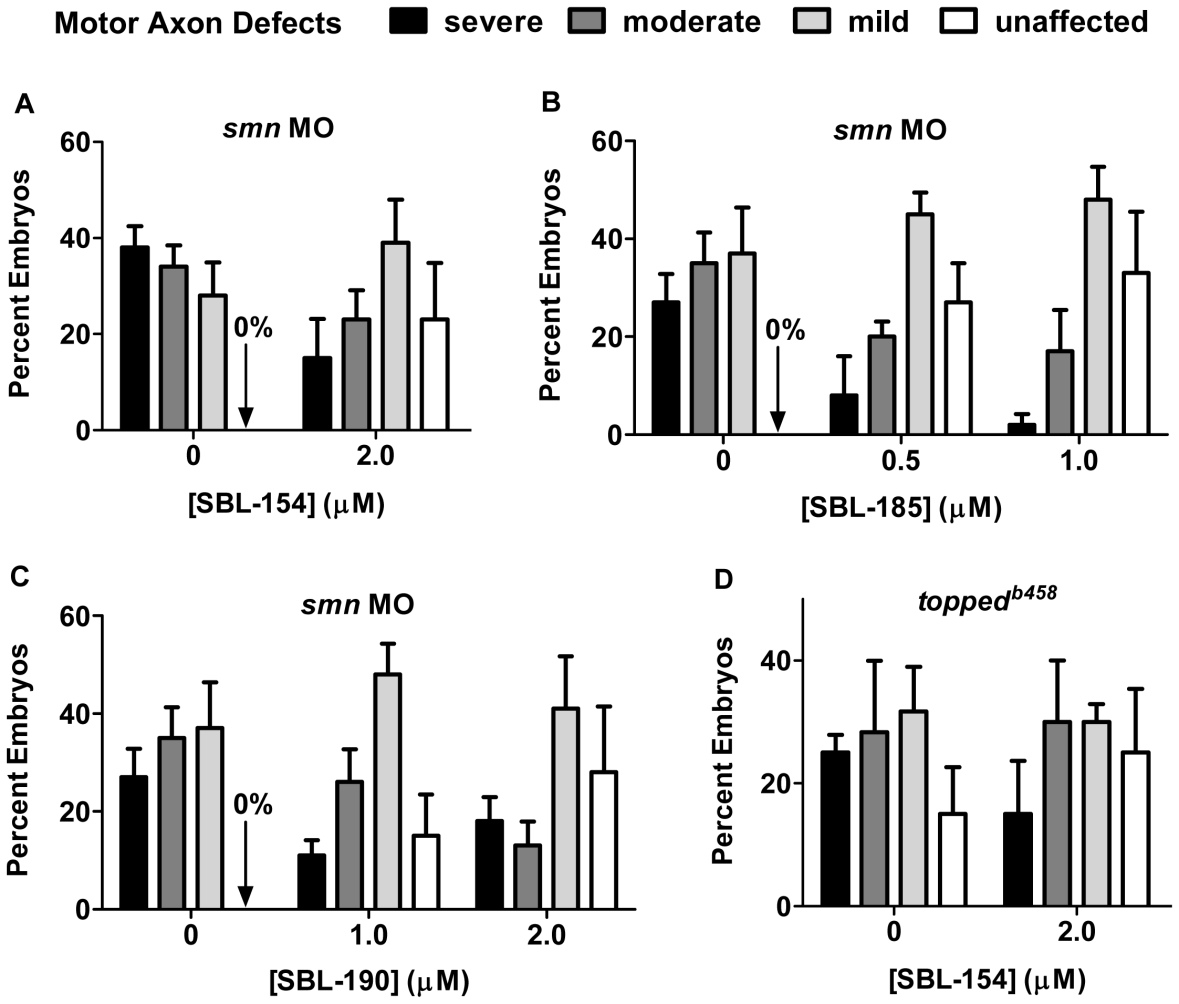
Rescue of motor axon development by axonotrophic compounds; combined results. (A) The results with 2 µM SBL-154 were averaged across the experiments in Figure 7A–F for both the control (vehicle-treated embryos) and experimental conditions, and mean values are plotted along with SEM (N = 6). SBL-154 significantly reduced the severity of motor axon defects (p<0.0001, N = 6; Mann-Whitney U test). (B) Suppression of Smn knockdown by SBL-185 was tested in 5 experiments, each of which examined 3 conditions: 0.5 µM SBL-185 (95 embryos scored among the 5 experiments, with about 20 embryos per experiment), 1 µM SBL-185 (95 embryos), and DMSO vehicle (99 embryos). Both concentrations of SBL-185 significantly reduce the severity of motor neuron dysmorphism (p<0.0001, N = 5; Kruskal-Wallis one-way ANOVA). (C) Suppression of Smn knockdown by SBL-190 was tested in 5 experiments, each of which examined 3 conditions: 1 µM SBL-190 (88 embryos), 2 µM SBL-190 (88 embryos), and DMSO vehicle (99 embryos). Both concentrations of SBL-190 significantly reduce the severity of motor neuron dysmorphism (p<0.0001, N = 5; Kruskal-Wallis one-way ANOVA). (D) Suppression of motor axon defects in *topped^b458^* mutants by SBL-154 was tested in 3 experiments in which mutants were treated with either DMSO (60 embryos) or 2 µM SBL-154 (60 embryos). SBL-154 showed no effect (p = 0.14; Mann-Whitney U test).

We first tested the ability of SBL-154 to rescue normal development of motor axons in Smn-deficient embryos. Figures 7A–F show results of the 6 experiments in which embryos were injected with *smn* MO and allowed to develop in fish water containing either DMSO vehicle or compound from 10 to 28 hours post fertilization (hpf). In 4 of the 6 experiments, exposure of embryos to 2 µM SBL-154 resulted in a statistically significant redistribution of embryos among the severe, moderate, mild, or unaffected classes. Even in the two experiments in which treatment with SBL-154 failed to significantly change the distribution, the compound mitigated, albeit non-significantly, the extent of motor axon defects. Indeed, results averaged from the six experiments indicate that the compound significantly suppressed the defects in motor axon development (Fig. 8A). The compound’s effects are dramatically illustrated by considering embryos that were unaffected by Smn knockdown: among the 6 experiments, none of the 131 embryos exposed to DMSO vehicle were completely free of motor axon defects; in contrast 25% of compound-treated embryos (32 of 126) were unaffected.

To look at these data in more detail, we constructed a metric, Motor Axon Defect Score, to quantify the severity of motor axon defects (see Materials and Methods). Motor Axon Defect Scores, in turn, were used to calculate the effectiveness of candidate suppressors, which we express as Fractional Rescue (see Materials and Methods). Fractional Rescue of 1 indicates the absence of any motor axon defects among compound-treated Smn-deficient embryos (that is, full rescue); Fractional Rescue of 0 indicates equal motor axon defects between vehicle-treated and compound-treated Smn-deficient embryos (that is, no rescue). We used the Fractional Rescue to determine whether rescue was correlated with the dose of MO or with the severity of the motor axon defects. The nominal amount of *smn* MO injected into embryos varied from 6 to 9 ng among the experiments of Figures 7A–F. Figure 7G plots the Fractional Rescue for SBL-154 as a function of the amount of *smn* MO that was delivered in each experiment. Although there is a suggestion of an inverse correlation between the suppressor effect of SBL-154 and the nominal amount of *smn* MO delivered, the slope of the linear regression is not significantly different from zero (p = 0.07). Several factors, such as aging-dependent changes in MO concentration and differing efficacies among batches of MO, could obscure a relationship between suppression and the amount of MO delivered. A far more significant association exists between the suppressor effect of SBL-154 and the Motor Axon Defect Score of vehicle-treated embryos (Fig. 7H). This analysis indicates that 97% of the variance in the suppressor effect of SBL-154 among the experiments in Figure 7A–F can be attributed to various severities of *smn* MO-induced motor axon defects. Importantly, the analysis also indicates that the greater the severity of the *smn* MO-induced motor axon defects, the less effective the compound is in suppressing those defects.

SBL-185 and SBL-190, the two analogs of SBL-154 that likewise promote neurite outgrowth of cultured spinal motor neurons (Fig. 6), were also tested for rescue of Smn knockdown. SBL-190 was tested at both 1 and 2 µM; SBL-185, because of its lower maximum nontoxic dose in zebrafish, was tested at 0.5 and 1 µM. As shown in Figures 8B–C, both compounds significantly rescued motor axon development. Again, the suppressor effects of the compounds are clearly illustrated by considering the MO-injected embryos that were completely free of motor axon defects: no such unaffected embryos were among the 99 embryos exposed to DMSO vehicle; in contrast 30% of embryos treated with 1 µM SBL-185 (29 of 95) and 31% of embryos treated with 2 µM SBL-190 (27 of 88) were free of motor axon defects. Taken together, these data show that all three compounds alleviate the motor axon defects caused by low levels of Smn.

To determine whether the suppressor activities of the compounds were specific to defects caused by low levels of Smn, we tested the effect of a representative compound, SBL-154, on motor axons in *topped^b458^* mutant zebrafish [62]. Like Smn-deficient zebrafish, *topped^b458^* mutants exhibit aberrant CaP motor axons during the first day of development, but for different reasons: while aberrant motor axon morphology in *smn* MO-treated embryos is due to low Smn levels, dysmorphic motor axons in *topped^b458^* mutants likely result from reduced levels of Semaphorin 5A [63], [64]. As shown in Figure 8D, SBL-154 had no effect on the motor axon defects in *topped^b458^* mutants, which is consistent with the hypothesis that the compounds’ effects are specific to low levels of Smn.

**Figure 9:**
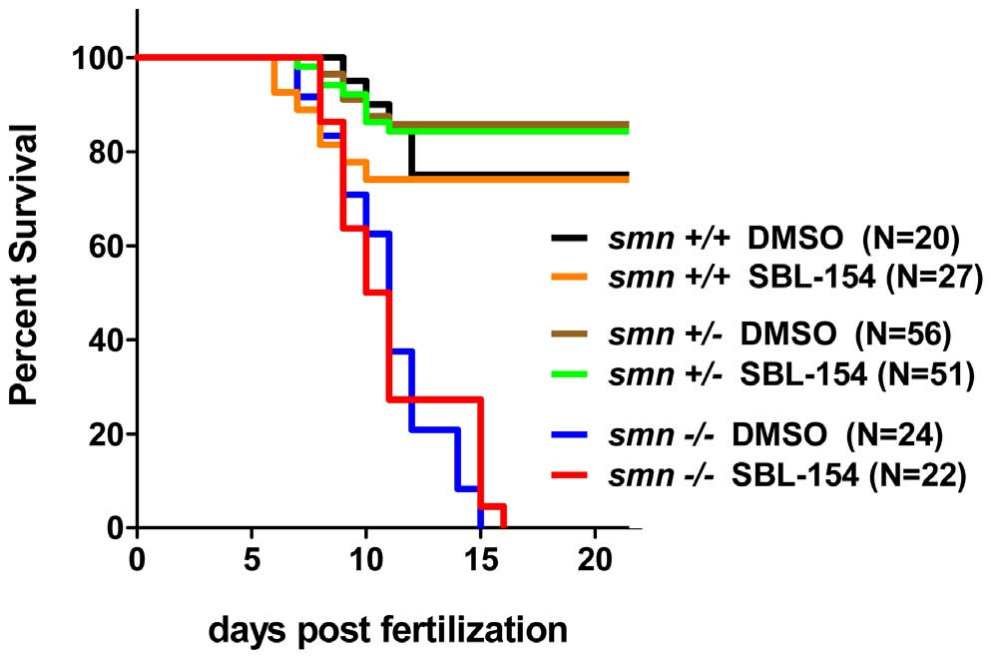
Failure of SBL-154 to extend lifespan of *smn −/−* zebrafish larvae. Larval progeny of *smn +/−* parents were treated with 2 µM SBL-154 or DMSO vehicle. Kaplan-Meier survival curves for the genotyped larvae are shown. The log-rank test for statistical significance indicates no difference between DMSO- and SBL-154-treated larvae for any of the three genotypes (p = 0.77, 0.84, and 0.52 for the wild-type, heterozygote, and mutant larvae, respectively).

We next evaluated whether the suppressor compounds of this study affect survival. Specifically, we tested the effects of SBL-154 on survival of *smn −/−* progeny of *smn +/−* heterozygous parents. As shown in Figure 9, SBL-154 had no effect on the survival of the *smn −/−* larvae, suggesting that the compound’s ability to rescue motor axon defects is not sufficient to increase survival.

### The Compounds behave as γ-Secretase Modulators

**Figure 10:**
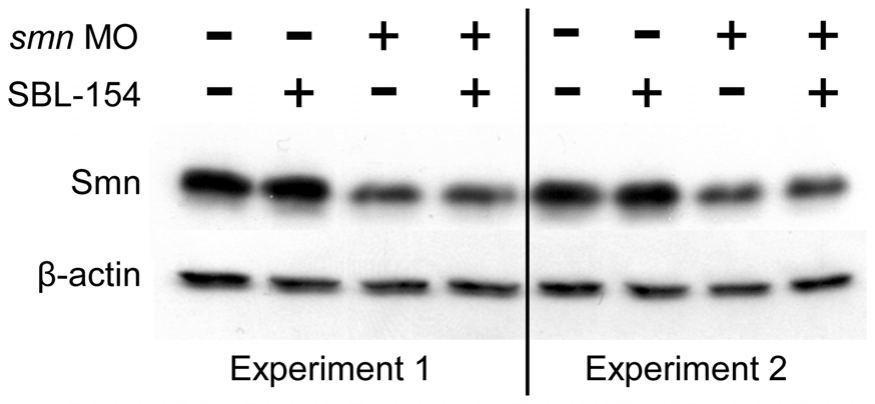
Lack of effect of SBL-154 on Smn protein levels. Zebrafish embryos not injected with morpholino (−) or injected with 9 ng *smn* MO (+) were treated with either DMSO vehicle (−) or 2 µM SBL-154 (+). Approximately 25 embryos of each of the four classes were pooled, and samples of each class were analyzed by Western blot for Smn protein. β-actin levels were used to normalize protein loading. SBL-154 had no effect on levels of Smn protein in either the presence or absence of *smn* MO.

The compounds that rescued motor axon development in the zebrafish SMA model could act by raising Smn protein levels. They could, for example, interfere with the morpholino-mediated knockdown of Smn or increase expression of Smn as has been shown for inhibitors of GSK-3 [65]. We addressed this possibility by Western blot analysis of Smn levels in embryos treated with SBL-154. As shown in Figure 10, morpholino-mediated reductions in Smn levels were not affected by SBL-154. These data indicate that the action of the three compounds in suppressing motor axon defects is independent of Smn levels.

The compounds that rescued motor axon development in the zebrafish SMA model were disclosed in the patent literature as modifiers of Aβ metabolism [36]. A likely target of the compounds is therefore γ-secretase, the enzyme complex responsible for production of Aβ peptides by proteolysis of APP. Since Notch, a protein critical for tissue development and homeostasis [66], is another substrate of γ-secretase, compounds that target γ-secretase can have teratogenic or carcinogenic effects [67]. For this reason developers of pharmaceuticals for Alzheimer’s disease have expended enormous efforts to identify compounds that modify γ-secretase-mediated processing of APP without affecting the enzyme’s processing of Notch [68]. The pediatric nature of SMA magnifies concerns of our compounds’ potential for detrimental effects on development.

The formation of somite borders in zebrafish larvae requires proper processing of Notch by γ-secretase, and the regularity of these somite boundaries provides a sensitive, *in vivo* indicator of accurate Notch processing [69]–[72]. At the concentrations that rescued motor axon development in Smn-deficient embryos, we found no effect of SBL-154, SBL-185, or SBL-190 on the somite borders of normal larvae. Indeed, irregular somite boundaries were never observed at concentrations below their toxic thresholds, which are at least twice the concentrations tested on Smn-deficient embryos (data not shown). In contrast, treatment with 32 µM DAPT, which has been shown to alter Notch processing in zebrafish [73], consistently disrupted the regularity of somite boundaries. Thus, the compounds’ rescue of motor axon development is not associated with altered Notch processing.

Agents that discriminately modify the processing of APP by γ-secretase without affecting its activity on Notch have been designated γ-secretase modulators [74]. γ-secretase modulators are thus pharmacologically distinct from conventional γ-secretase inhibitors such as DAPT, which affects processing of all γ-secretase substrates. If SBL-154, SBL-185, and SBL-190 were γ-secretase modulators, it would reconcile the compounds’ inactivity on Notch processing in zebrafish with their disclosed effects on Aβ metabolism. It would also rationalize the results shown in Figure 2, where the compounds and DAPT have qualitatively different effects on locomotion of kinesin-deficient *Drosophila* larvae. In summary, our observations are consistent with the suppressor compounds behaving as γ-secretase modulators. In fact, a defining characteristic of the compound collection from which SBL-154, SBL-185, and SBL-190 were drawn is that its members behave as γ-secretase modulators (B.F.X. Dowd [Myriad Pharmaceuticals, Inc.], personal communication).

## Discussion

Using motor neuron assays from both vertebrates and invertebrates, we explored the activities of a number of chemical analogs that had been disclosed in the patent literature as modifiers of Aβ metabolism. An accounting of the compounds tested, assays used, and results obtained in this study are presented in Tables 1 and 2. Collectively the data indicate that the compounds promote motor axon growth and function. Of particular relevance to motor neuropathies, three of the compounds rescued motor axon development in a zebrafish model of SMA. Our findings nominate the three small molecules for development as candidate SMA therapeutics, reinforce the perception of SMA as an axonopathy, and encourage the exploration of γ-secretase modulators as pharmacologic tools to probe motor neuropathology.

**Table 1:** Compounds tested and results obtained in *Drosophila*, enzymatic, and cultured motor neuron (MN) assays in this study.

	*Drosophila*			Enzymatic	Primary Spinal MNs	
Agent	LocomotionRescue of*khc/+; klc/+*	AggregateReduction in*khc/+; klc/+*	Level of Agent in Hemolymph	Purified GSK3β Inhibition	Neurite Outgrowth Stimulation	Motor Neuron Survival
SBL-154	active	×	×	×	active	no effect
SBL-185	×	×	×	×	active	no effect
SBL-190	×	×	×	×	active	no effect
SBL-398	active	active	228 nM	no effect	x	x
SBL-429	active	×	×	×	×	×
SBL-776	active	×	×	×	×	×
SBL-897	no effect	×	×	×	×	×
DAPT	no effect	×	×	×	×	×
AR-A014418	×	×	×	active	×	×
BDNF	×	×	×	×	active	active
DMSO	no effect	no effect	0 nM	×	no effect	no effect

× indicates that the agent was not tested in the specified assay.

**Table 2:** Compounds tested and results obtained in zebrafish assays in this study.

Agent	Suppression of*smn* MO	Disruption of Somite Borders	Changes in Smn Protein Levels	*topped43^b458^* Suppression	Survival of *smn−/−*
SBL-154	active	no effect	no effect	no effect	no effect
SBL-185	active	no effect	×	×	×
SBL-190	active	no effect	×	×	×
SBL-398	×	×	×	×	×
SBL-429	×	×	×	×	×
SBL-776	×	×	×	×	×
SBL-897	×	×	×	×	×
DAPT	×	active	×	×	×
AR-A014418	×	×	×	×	×
BDNF	×	×	×	×	×
DMSO	no effect	no effect	no effect	no effect	no effect

× indicates that the agent was not tested in the specified assay.

### Axonotrophic Activities of Compounds

*Drosophila* larvae that are deficient in functional kinesin are relevant to human motor neuropathies. Mutations in *Kif5A*, the human ortholog of *Drosophila Khc*, can cause both Charcot-Marie-Tooth Type 2 disease (CMT2) and the SPG10 form of hereditary spastic paraplegia (HSP) [75]–[79]. Like kinesin-deficient *Drosophila*, patients who suffer from CMT2 or the SPG10 form of HSP exhibit dystrophic axon terminals, reduced axonal transport, and accumulation of axonal aggregates, all of which precede distal neuropathy [35], [79]–[82]. Clearly, kinesin-deficient *Drosophila* models these diseases. But the relevance of kinesin-deficient *Drosophila* to motor neuropathies extends beyond diseases caused by mutant kinesin to additional diseases in which axonal transport is compromised and axonal swellings are observed. These diseases include amyotrophic lateral sclerosis (ALS) [83], [84], Huntington’s disease [85], [86], Parkinson’s disease [87], [88], forms of HSP in addition to SPG10 [82], [89], and SMA [90]–[92]. Small molecules that suppress the larval locomotion defect of kinesin-deficient *Drosophila* therefore merit evaluation as candidate therapeutics for such motor neuropathies.

Our data demonstrate that SBL-398 suppresses the locomotion defect of kinesin-deficient *Drosophila* larvae, reduces the load of axonal aggregates that correlate with the motor dysfunction, achieves these effects at pharmacologically reasonable concentrations *in vivo*, and has no inhibitory activity versus GSK-3β (Figs. 2–5). Moreover, three analogs of SBL-398 likewise suppressed the locomotion defect of *khc/+; klc/+* larvae, suggesting that the four chemically related modifiers of Aβ metabolism promote the function of motor axons. Among the compounds that suppressed the uncoordinated locomotion of *khc/+; klc/+ Drosophila* larvae, one compound (SBL-154) has attractive PK and ADMET properties in both rats and mice (data not shown). SBL-154 enhanced neurite outgrowth in embryonic rat spinal cord motor neurons, demonstrating that its suppression of motor dysfunction in *Drosophila* is accompanied by growth-promoting effects on mammalian motor neurons. Two analogs of SBL-154, each resembling SBL-154 in its effect on Aβ production and its drug-like PK/ADMET properties, also stimulated motor axon outgrowth. Since none of the three compounds consistently increased survival of the cultured spinal motor neurons, we designate them as axonotrophic instead of neurotrophic agents to best reflect their biological effects.

### Biological Significance of Suppressor Activities in the Zebrafish Model of SMA

The three axonotrophic compounds that we have identified also significantly suppressed motor axon defects in a zebrafish model of SMA. To our knowledge, this is the first report of effective pharmacologic suppression in this SMA model. The question remains, though, whether the compounds are sufficiently active in this zebrafish model of SMA to justify their further evaluation as candidate SMA therapeutics. We assessed the biological significance of the compounds’ effects by referencing a previous study that used the same zebrafish model to evaluate the comparative suppressor activities of human SMN alleles [60]. That study found that motor axon abnormalities resulting from *smn* MO injection were significantly suppressed by co-injection of RNA encoding wild-type SMN. From the published results we calculate a Fractional Rescue of 0.58 for injection of RNA encoding wild-type SMN. The Fractional Rescues of 2 µM SBL-154, 1 µM SBL-185, and 1 µM SBL-190 reported herein averaged 0.43, 0.54, and 0.37, respectively. Experimental differences between the published genetic study and our pharmacologic study preclude quantitative comparisons between the two. For example, the mosaic nature of RNA injection, whereby RNA levels can vary among the different cells of the injected embryo, can obscure the full effect of the RNA treatment. Also, as we have shown here, different severities of Smn knockdown can influence suppressor effects. While such experimental differences prevent quantitative comparisons, we nonetheless interpret the Fractional Rescue values as a qualitative indication that rescue by the compounds is comparable to rescue by SMN itself and thus is biologically meaningful.

Yet a compound (SBL-154) that effectively suppressed motor axon defects in zebrafish with morpholino-mediated reductions in Smn had no effect on the survival of *smn −/−* larvae (Fig. 9). The different efficacies of SBL-154 in the morpholino and genetic models of SMA may indicate that the compound’s therapeutic effect is restricted to motor neurons, since previously reported suppressor strategies that target motor neurons also failed to increase survival [93], [94]. Even increased SMN levels – if restricted to motor neurons – have limited effects on survival of zebrafish and mouse models of SMA [95]–[98], which is consistent with the emerging view of SMA as multisystem disorder [99], [100]. Alternatively, the different efficacies of SBL-154 in the morpholino and genetic SMA models may reflect different degrees of Smn deficiencies in the two models. When severe motor axon defects were achieved with high concentrations of *smn* MO, SBL-154 failed to suppress the defects (Fig. 7). In the genetic model, a rapid decay of maternally transferred Smn may expose *smn −/−* embryos to Smn deficiencies that likewise cannot be suppressed by the compound. Finally, it is possible that the different timings of exposure to SBL-154 (continuously from 10 to 28 hpf *versus* 4 hours daily from 4 to 10 dpf) account for its different efficacies in the morpholino and genetic models.

### Implications of Results with the Zebrafish Model of SMA

Since SMA is a consequence of insufficient SMN protein, agents that increase SMN levels have been the focus of extensive developmental efforts [101], [102]. There have been fewer reports of therapeutic strategies that suppress the SMA phenotype, yet these approaches have also shown promise [102]. Particularly notable is the demonstration that pharmacologic inhibition of ROCK dramatically prolongs survival in a mouse model of SMA [103], [104]. The compounds we report here provide an alternate, independent opportunity to explore phenotypic suppression as a strategy for SMA treatment. Since the compounds are new chemical entities never before reported in the scientific literature, they define a novel pharmacologic approach to treating SMA. Also, the compounds show attractive PK and ADMET properties in mice (data not shown). Thus, the distinguishing character of compounds as pharmacologic suppressors, their novel chemical structures, and their drug-like PK/ADMET properties recommend them for testing in available mouse models of SMA.

In addition to nominating three particular small molecules for testing in mouse models of SMA, these results advise exploring two specific classes of agents as SMA therapeutics. The first class consists of agents that promote the growth and function of motor axons. An early event in expression of the SMA phenotype is denervation of neuromuscular junctions of clinically relevant muscles due to failure of synaptic maintenance at their motor end plates [105]–[109]. Accordingly, interventions that either support synaptic maintenance before frank denervation has occurred or promote growth of axonal sprouts in the early stages of denervation may effectively suppress disease progression. The results reported herein encourage the identification of additional axonotrophic agents that act on motor neurons and the testing of those agents as possible SMA therapeutics.

The second class of agents that may prove to be effective as SMA treatments consists of γ-secretase modulators, a structurally and mechanistically diverse group of compounds that alter γ-secretase-mediated proteolysis of a subset of its substrates [68], [74]. The three SMA suppressors reported here were reported to inhibit production of the APP-derived peptide Aβ42 [36], thus suggesting that inhibition of γ-secretase accounts for their suppressor activities. Yet the suppressors failed to affect γ-secretase-mediated processing of Notch in zebrafish, and their effects on kinesin-deficient *Drosophila* larvae were not shared by the general γ-secretase inhibitor DAPT. These apparently discordant observations are consistent with the hypothesis that the suppressors are γ-secretase modulators, an inference for which others have found experimental support (B.F.X. Dowd [Myriad Pharmaceuticals, Inc.], personal communication). Our results therefore recommend testing additional γ-secretase modulators as candidate SMA therapeutics. Indeed, given the structural and mechanistic heterogeneity of γ-secretase modulators [74], such tests would constitute a rigorous evaluation of the hypothesis that γ-secretase is a therapeutic target for SMA.

SMA is a devastating disease for which no drug treatment has been proven to have significant efficacy in clinical studies [101]. Our hope is that the results reported here will instruct the exploration of novel therapeutic approaches that are distinct from and may complement the on-going development of agents that increase SMN protein levels [102]. Given the commonalities of motor neuropathies like SMA and ALS [110], such novel approaches may also advance the development of therapeutics for other motor neuropathies.

## Materials and Methods

### Ethics Statement

The zebrafish experiments were conducted at The Ohio State University and the University of Utah in accordance with recommendations in the Guide for the Care and Use of Laboratory Animals of the National Institutes of Health under protocols 2009A0141 (OSU) and 10–11002 (UU), which were approved by the universities’ Institutional Animal Care and Use Committees. The neurite outgrowth and survival assays were conducted under contract by Neurofit SAS (Illkrich, France), which holds relevant institutional and project licenses from the French government. All procedures used in this study conformed to the French Animal Health Regulation, the NIH Guide for the Care and Use of Laboratory Animals, the Recommendations for Euthanasia of Experimental Animals issued by the European Commission, and the recommendations of the American Veterinary Medical Association (AVMA) Guidelines on Euthanasia.

### Synthetic Small Molecules

Compounds SBL-154, SBL-185, SBL-190, SBL-398, SBL-429, SBL-776, and SBL-897 were synthesized and purified to >90% purity as described [36]. AR-A014418 (also known as GSK-3β Inhibitor VIII) and DAPT (N-[N-(3,5-difluorophenacetyl-L-alanyl)]-S-phenylglycine t-butyl ester) were purchased from EMD Millipore. Structures are shown in Figure 1.

### Construction of *Drosophila* Strains

Reductions of kinesin function in *Drosophila* can result in a larval locomotion defect characterized by a rhythmic elevation of the tail due to paralysis of muscles in ventral posterior segments [111], [112]. The phenotype is observed in animals with mutations in either the heavy or the light chains of kinesin-1 (formerly termed conventional kinesin), encoded by *Khc* and *Klc*, respectively. The severity and penetrance of the phenotype depends on the combination of mutant alleles carried by the larvae [113]. Thus, *khc/+; klc/+* double heterozygotes exhibit phenotypes that are intermediate between *khc/khc* or *klc/klc* homozygotes and single *khc* or *klc* heterozygotes. Furthermore, the severity of the phenotype of *khc/+; klc/+* double heterozygotes depends on the particular alleles of *Khc* and *Klc*. We constructed a *khc/+; klc/+* heterozygote that exhibits a penetrance of approximately 70%; we presumed that the severity of the phenotype would be sufficiently low to be pharmacologically suppressible yet sufficiently high to allow discrimination of rescues with different efficacies. Such an intermediate penetrance also allows the formal possibility of identifying agents that exacerbate the locomotion defect. Thus, publicly available stocks of *b* [*1*] *pr* [*1*] *Khc* [*8*] */CyO cy* and *y* [*1*] w[*]; T(2∶3)B3 CyO; TM6B Tb [*1*] */Pin[88K]* were used to obtain female *b* [*1*] *pr* [*1*] *Khc* [*8*] *; T(2∶3)B3 CyO; TM6B Tb* [*1*] that were mated to male w[*]; Df(3L)8ex25/TM6B Tb [*1*] animals. The female wild-type *Khc* allele can be followed using the larval marker Tubby to distinguish it from the amorphic *Khc* [*8*] (http://flybase.org). The male wild-type *Klc* allele, which encodes the kinesin light chain, is also marked by Tubby, allowing it to be distinguished from the deletion that includes the *Klc* locus. The *b* [*1*] *pr* [*1*] *Khc* [*8*] *; Df(3L)8ex25* larval progeny from this cross, identified by their normal (non-tubby) body shape, are the experimental *khc/+; klc/+* double heterozygotes that are scored in this study. Fly stocks were crossed and maintained at 25^o^C; phenotypic assays were scored at room temperature.

### Scoring of *Drosophila* Motor Phenotype

Experimental larvae were raised on Instant Drosophila Medium (Carolina Biological Supply, Burlington, NC) supplemented with 0.1% bromophenol blue and containing dimethyl sulfoxide (DMSO) alone or test compounds dissolved in the DMSO vehicle. None of the compounds had a discernible effect on larval development at the concentrations reported herein. The locomotion phenotype of non-tubby wandering third instar larvae was scored as either wild-type or uncoordinated, based on visual detection of the characteristic tail-flipping exhibited by kinesin-1 mutants [35]. The person who scored the locomotion phenotype was unaware of the compound treatment of the animals. No wild-type larva was seen to exhibit the tail-flipping phenotype.

### Measurements of Intraneuronal Aggregates

Immunostaining of segmental nerves of *Drosophila* larvae was performed as described [35]. Experimental larvae were grown on Instant Drosophila Medium containing 0.5mM SBL-398 or DMSO vehicle until the wandering third instar stage, when they were dissected in calcium-free buffer (128 mM NaCl, 2 mM KCl, 1 mM EGTA, 4 mM MgCl_2_, 5 mM HEPES (pH 7.1)) and fixed in several changes of 4% paraformaldehyde. Larval pelts were then permeabilized with several washes of PBS (137 mM NaCl, 2.7 mM KCl, 10 mM Na_2_HPO_4_, 2 mM KH_2_PO_4_, pH7.4) containing 0.1% Triton X-100; stained overnight at 4°C with rabbit anti-synaptotagmin (Santa Cruz Biotechnology, Santa Cruz, CA); labeled with fluorescein (FITC)-conjugated anti-rabbit antibody (Molecular Probes, Eugene, OR); and mounted for confocal microscopy. Synaptotagmin labels synaptic vesicles, which can accumulate at sites within dysfunctional axons where membranous material is deposited to form structures that are variously termed axonal swellings, axonal spheroids, and neuritic beads. These synaptotagmin-marked, intraneuronal aggregates were imaged using FluoView® software on an Olympus FV1000 confocal laser scanning microscope (Olympus, Center Valley, PA). Images were taken from segmental nerves passing through larval segment A4 for standardization. A 60× oil immersion objective was used along with a 488 nm excitation laser optimized for detection of the FITC fluorophore. Z-series stacked images were obtained at a step size of 0.5 µm over a 10–30 µm range for each field. Raw z-stacks were then processed using Volocity® 3D Image Analysis Software (v 2.5, Improvision (PerkinElmer) Waltham, MA) to render 3-D images and calculate the volumes of synaptotagmin-positive objects. Only individual, distinct swellings that were also larger than 1 µm^3^ were processed.

### Measurements in Hemolymph

Wild-type *Drosophila* larvae were raised on media containing 0.5 mM SBL-398 or DMSO (vehicle). Wandering third instar larvae that had recently (within 60 minutes) climbed from the media were collected, washed three times in PBS containing 0.1% Tween 20 to remove compound that may adhere to the cuticle, and rinsed three times in PBS to remove both any remaining compound and residual Tween 20 from the washes. Washing steps consumed less than 20 minutes in total. Larvae were then dissected individually for collection of about 0.25 µl hemolymph per animal. Since their food is stained with bromophenol blue, larvae could be dissected without damaging the clearly visible gut, thus avoiding contamination of collected hemolymph with intestinal contents. The visible presence of food in the gut indicated that a reservoir of compound- or vehicle-containing media existed within the gut throughout the procedure. Approximately 50 µl of hemolymph were collected from experimental and from control larvae, diluted to 200 µl in PBS, and stored at −20C until thawed for mass spectroscopic analysis. This was done with independent duplicate samples for both the experimental and control conditions, yielding a total of 4 samples that represented about 800 dissected larvae. Hemolymph samples were fortified with an internal standard, extracted with ethyl acetate, reconstituted, and subjected to liquid chromatography-tandem mass spectrometry (LC-MS/MS) analysis as follows. Extracted samples were injected onto a reversed phased liquid chromatography system (Shimadzu LC-10 with an Agilent Zorbax C-18, 50 mm×4.6 mm column) serving an AB Sciex API4000 QTrap mass spectrometer. The mass spectrometer operated a multiple reaction monitoring method in positive ion mode with an electrospray ionization source. Synthetic standards were used to generate a calibration curve and quality control samples in a PBS surrogate matrix. The quantitation range was 1–1000 ng/ml based on the analysis of 50 µl of diluted hemolymph. Back-calculated values for each calibration standard and quality control sample were within 15% of the theoretical concentration, and the coefficients of determination for the calibration curves were >0.99.

### GSK-3β Assay

Human GSK-3β (GenBank Accession Number NP_002084.2) was purchased as an N-terminally His-tagged protein expressed in Sf9 cells (Sigma-Aldrich Catalog Number G4296; reported purity ≥70%). Assays were performed as recommended by supplier: at 30°C in a final medium of 3 mM MOPS (pH 7.2), 1.5 mM glycerol 2-phosphate, 3 mM MgCl_2_, 0.6 mM EGTA, 0.24 mM EDTA, 0.03 mM dithiothreitol, and 20 µg/ml BSA. The enzymatic assay measured γ-^32^P-ATP labeling of peptide substrate YRRAAVPPSPSLSRHSSPHQSEDEEE, with a ratio of unlabeled to labeled ATP (1 mCi/100 µl; Amersham Pharmacia Biotech) of 1.5. We determined the activity of 4 nM GSK-3β at ATP concentrations of 0, 2.5, 8.0, and 25 µM for each of the following 6 conditions: peptide substrate concentrations of 0.8, 2.4, and 7.2 nM and reaction times of 30 and 60 minutes. All conditions yielded equivalent values of Km(ATP), which averaged 24±4.5 µM (mean ± SD, N = 6), acceptably close to the expected 15 µM. Inhibition of this GSK-3β activity by SBL-398 and AR-A014418 was therefore assayed over 30 minutes with [GSK-3β = 4 nM, [peptide substrate] = 12 nM, and [ATP] = 30 µM. Concentrations of SBL-398 and AR-A014418 were tested up to 25 and 50 µM, respectively.

### Rat Motor Neuron Survival and Neurite Outgrowth

Pregnant Wistar rats were sacrificed at 15 days gestation by cervical dislocation, fetuses were removed, and fetal spinal cords were dissected into ice-cold medium of Leibovitz (L15, Gibco), where their meninges were carefully removed. The spinal cords were dissociated by treatment with trypsin (Gibco) for 30 minutes at 37°C in the presence of DNAse I (Boehringer Mannheim, France); proteolysis was terminated by addition of DMEM containing 10% fetal bovine serum (Gibco). The suspension was triturated using a 10 ml pipette and a needle syringe followed by centrifugation at 580×g for 10 min at room temperature (RT). The pellet of dissociated cells was resuspended in L15 medium, and the resulting suspension was centrifuged for 10 min at 180×g at RT on a layer of 3.5% solution of bovine serum albumin in L15 medium. The supernatant was discarded, the pellet was resuspended in L15 supplemented with 1% DNAase I, the suspension was layered on a cushion of Optiprep® (Abcys, France), and the preparation was centrifuged at 400×g for 25 min at RT. The upper phase, containing purified spinal motor neurons, was collected, resuspended in L15, and centrifuged at 800×g for 10 min at RT. The cell pellet was finally resuspended in a defined culture medium consisting of Neurobasal Medium® (Gibco) supplemented with 2% B27® Supplements (Gibco) and 5 mM L-glutamine (Gibco). Viable cells were counted in a Neubauer cytometer using the Trypan Blue exclusion test (Sigma-Aldrich). For neurite outgrowth assays, 30,000 purified rat spinal motor neurons were seeded on 35 mm dishes (Nunc) coated with poly-L-lysine, allowed to adhere for 2 hours, and treated for three days with compounds, BDNF as positive control, or DMSO vehicle at 37°C in a humidified incubator with 5% CO_2_-95% atmospheric air. The length of the longest unbranched neurite was determined for each of ∼80 neurons for each condition. The 13 kd neurotrophin BDNF was tested at a concentration (3.7 nM) that has near-maximal effects on neurite outgrowth. To measure basal survival of the purified motor neurons, purified cells were seeded onto 96-well plates coated with poly-L-lysine at 4,000 cells per well, allowed to adhere, and treated with compound as described above. After 3 days of treatment with compounds, cell survival was evaluated using the acid phosphatase activity assay. Briefly, after removal of the culture medium the wells were rinsed with 100 µl PBS, and 100 µl 0.1 M sodium acetate (pH 5.5), 0.1% Triton X100, and 10 mM p-nitrophenyl phosphate (Sigma-Aldrich) was added. The development was stopped with 10 µl 1 N NaOH. Enzyme activity was measured at 405 nm in a microplate reader (Labsystems Multiskan Bichromatic). BDNF (3.7 nM) served as positive control.

### Zebrafish Motor Axon Morphology

*D. rerio* zebrafish, larvae, and embryos were maintained at 28.5°C and staged by hours post-fertilization (hpf) [114]. Transgenic *Tg(mnx1∶0.6hsp70:GFP)os26* embryos that express GFP in ventrally projecting motor axons [115], referred to as *Tg(mnx1:GFP)* embryos, were used for all knockdown experiments. Specifically, using an MPPI-2 Pressure Injector (Applied Scientific Instrumentation, Eugene, OR) and according to previous protocols [60], *Tg(mnx1:GFP)* embryos were injected at the one- to two-cell stage with antisense oligonucleotide CGACATCTTCTGCACCATTGGC (MOs; Gene Tools, Philomath, OR) to knock down Smn as previously described [59], [116], [117]. At 10 hpf injected embryos were placed in egg water (60 µg/ml Instant Ocean® sea salts) containing compound or DMSO (0.25%) vehicle and incubated at 28.5°C. To visualize motor axons in GFP transgenic animals, *Tg(mnx1:GFP)* embryos at 28 hpf were anesthetized with tricaine and fixed overnight at 4°C in 4% formaldehyde/PBS. After removing embryos from fix, their yolks and heads were removed and their trunks were mounted on glass coverslips for observation under a Zeiss Axioplan microscope. Motor axons innervating the mid-trunk (myotomes 6–15) on both sides of the fish were scored as described [60], which allowed each embryo to be classified as severe, moderate, mild, or unaffected according to previously described criteria based on the number and types of motor axon abnormalities [8]. *topped^b458^* embryos [62], [63] were treated and fixed as described above for *Tg(mnx1:GFP)* embryos; the *topped* mutants, however, were processed for znp1 antibody labeling as described previously [118] to visualize motor axons for scoring as they did not have the *Tg(mnx1:GFP)* on the background.

### Selection of Test Concentrations in Zebrafish

Selection of concentrations of small molecules for treating MO-injected *Tg(mnx1:GFP)* embryos was guided by determinations of their maximum nontoxic concentrations (MTDs) in wild-type AB/Tübingen embryos and larvae. These MTDs were determined by exposing embryos to compound dissolved in egg water containing 0.25% DMSO or to the DMSO vehicle alone beginning at 4, 11, and 25 hpf and continuing exposure for 7 days; media was replaced with fresh media every 48 hours after initiation of exposure. The following parameters were monitored throughout the treatment period: morphology (gross body shape), viability (strength of escape reflex), growth (larval length), somite boundaries (regularity of borders between somites), swim bladder development (inflation of swim bladder), and pigmentation (level of pigmentation of yolk sac and yolk sac extension). Observed MTDs were not noticeably affected by dechorionation of the embryos. Based on these observations in wild-type embryos and larvae, MO-injected *Tg(mnx1:GFP)* embryos with intact chorions were treated for a total of 18 hours, from 10 to 28 hpf, with SBL-154 and SBL-190 at 2 µM and with the slightly less tolerated SBL-185 at 1 µM. SBL-185 and SBL-190 were also tested at 50% lower concentrations (*viz*., 0.5 and 1 µM, respectively). As expected from the toxicity testing in wild-type embryos, these concentrations appeared nontoxic to MO-injected *Tg(mnx1:GFP)* embryos.

### Metrics of Motor Axon Dysmorphism and Suppression in Zebrafish

Quantification of the rescue of motor axon development in Smn-deficient zebrafish embryos required two steps. First, we constructed a Motor Axon Defect Score (MADS), defined as the sum of the following three components: 4 times the percentage of embryos with severely defective motor axons (Se); 2 times the percentage with moderately defective motor axons (Mo); and the percentage with mildly defective motor axons (Mi). Thus, MADS = 4*Se +2*Mo+Mi. While we feel that such a geometric grading scale most accurately reflects the degrees of dysfunction underlying motor axon dysmorphism, all results and conclusions presented in this report remain valid if a linear scale is used instead. For the second step we defined Fractional Rescue (FR) as 1 minus the ratio of Motor Axon Defect Scores for experimental and control embryos, or FR = 1– (MADS_experimental_/MADS_control_). In this study the experimentals were MO-injected embryos treated with compounds; the controls were MO-injected embryos treated with vehicle alone, which was 0.25% DMSO.

### Zebrafish Survival

Embryos from an incross of *smn Y292stop/+* zebrafish [95] were collected into housing tubes at a density of 25 embryos per tube and immersed in 10 liter tanks at 28.5°C for survival studies. From 4 to 10 dpf the larvae-containing tubes were transferred daily to 200 ml beakers that contained 48 ml egg water with either 2 µM SBL-154 or DMSO vehicle; after 4 hours of exposure to compound or DMSO, the tubes were returned to the 10 liter tanks. Two hundred embryos were evenly split between the SBL-154 and DMSO treatment groups. Dead larvae were collected daily until day 21, at which time survivors were sacrificed. Larvae were genotyped as described [95].

### Western Blot Analysis of Smn Protein Levels

Zebrafish embryos injected with 9 ng *smn* MO at the one- to two-cell stage and uninjected embryos were treated from 10 to 24 hpf with DMSO or with 2 µM SBL-154. As shown in Figure 10, 9 ng *smn* MO yielded a readily observable reduction of Smn levels, which allows detection of compound-dependent restoration of Smn levels. In each of two experiments, samples were generated by boiling 25 identically treated embryos in 75 µl blending buffer (63 mM Tris (pH 6.8), 5 mM EDTA, 10% SDS), yielding 4 samples for each of the duplicate experiments. For the 8 samples, 10 µl (equivalent to 3 embryos) were added to 10 µl of sample buffer (100 mM Tris (pH 6.8), 0.2% bromophenol blue, 20% glycerol, 200 mM dithiothreitol) and run on a 10% polyacrylamide gel, blotted to nitrocellulose, probed with mouse anti-Smn (MANSMA12, a gift from Dr. G.E. Morris [119]), and detected by chemiluminescence of bound HRP-conjugated mouse antibody. Blots were stripped and re-probed with mouse anti-β-actin (Sigma-Aldrich).

### Statistics

Statistical analyses, described in the figure legends, were conducted with the aid of GraphPad Prism version 5.0. Error bars in all graphs represent SEM. For all results comparisons are made to the DMSO condition, and the threshold for statistical significance is α = 0.05.
